# Optimization of Membrane Protein TmrA Purification Procedure Guided by Analytical Ultracentrifugation

**DOI:** 10.3390/membranes11100780

**Published:** 2021-10-12

**Authors:** Dongdong Li, Wendan Chu, Xinlei Sheng, Wenqi Li

**Affiliations:** 1 Institute of Biomedicine, Tsinghua University, Beijing 100084, China; lidongdong@mail.tsinghua.edu.cn (D.L.); chuwendan@mail.tsinghua.edu.cn (W.C.); 2 National Protein Science Facility, Beijing 100084, China; 3 School of Life Sciences, Tsinghua University, Beijing 100084, China; 4 Beijing Advanced Innovation Center for Structural Biology, Tsinghua University, Beijing 100084, China; 5 Lewis Thomas Laboratory, Department of Molecular Biology, Princeton University, Washington Road, Princeton, NJ 08544, USA

**Keywords:** membrane protein, analytical ultracentrifugation, protein purification

## Abstract

Membrane proteins are involved in various cellular processes. However, purification of membrane proteins has long been a challenging task, as membrane protein stability in detergent is the bottleneck for purification and subsequent analyses. Therefore, the optimization of detergent conditions is critical for the preparation of membrane proteins. Here, we utilize analytical ultracentrifugation (AUC) to examine the effects of different detergents (OG, Triton X-100, DDM), detergent concentrations, and detergent supplementation on the behavior of membrane protein TmrA. Our results suggest that DDM is more suitable for the purification of TmrA compared with OG and TritonX-100; a high concentration of DDM yields a more homogeneous protein aggregation state; supplementing TmrA purified with a low DDM concentration with DDM maintains the protein homogeneity and aggregation state, and may serve as a practical and cost-effective strategy for membrane protein purification.

## 1. Introduction

Membrane proteins account for ~20–30% of eukaryotic proteome and play crucial roles in a wide variety of cellular functions, such as regulating transmembrane ion transport, sensing and transmitting chemical or electrical signals, mediating cellular attachment, and modulating membrane lipid composition. They are classified into integral and peripheral membrane proteins. Integral membrane proteins are embedded in the lipid bilayer, while peripheral membrane proteins are weakly associated with the hydrophilic surfaces of the lipid bilayer [1,2]. It is estimated that membrane proteins constitute more than 60% of current drug targets, and approximately ~35% of currently approved drugs target one class of membrane proteins, G protein-coupled receptors (GPCRs). Therefore, investigation of membrane proteins is crucial for biomedicine [3]. However, membrane proteins are heavily underrepresented in the protein data bank (PDB) due to the difficulties of purification, and the hydrophobic or amphipathic nature of membrane proteins also hinders the preparation of homogeneous and stable proteins for structural studies [4,5]. In the process of membrane protein purification, detergents are used to solubilize membrane proteins and to shield their hydrophobic surfaces in aqueous solutions. There are various choices of detergents, including, but not limited to, alkyl-maltosides and glucopyranosides (e.g., *n*-dodecyl-β-D-maltopyranoside, β-DDM and *n*-octyl β-D-glucopyranoside, β-OG), amine oxides (e.g., 3-laurylamido-N, N’-dimethylpopylaminoxide, LAPAO), ethylene glycols (e.g., dodecyl octaethylene glycol ether, C_12_E_8_ and α-[4-(1,1,3,3-tetramethylbutyl)phenyl]-ω-hydroxy-poly (oxy-1,2-ethanediyl)], Triton X-100), cholesterol (e.g., 3-[(3-Cholamidopropyl) dimethylammonio]-1-propanesulfonate hydrate, CHAPS), and the new class of neopentyl glycol, NG, most of which are costly. Therefore, it is critical to choose the appropriate type and concentration of detergent in the interest of cost and efficiency [6,7].

As the largest class of membrane proteins, ATP-binding cassette (ABC) transporters drive substrate translocation across membranes and modulate diverse important cellular processes, such as multidrug resistance in cancer cells, antibiotic resistance in bacteria, and common genetic diseases. A number of bacterial ABC exporters, including LmrA, MsbA, and BmrA, are homodimers, while most of the eukaryotic ABC exporters are heterodimers [8,9,10]. One such example is the heterodimer ABC transporter from *Thermus thermophiles*, TmrAB, which is responsible for drug extrusion. TmrAB was reported to exist in a finely-tuned equilibrium between inward- and outward-facing conformations. A previous study revealed the structure of TmrAB at a resolution of 2.7 -Å and demonstrated that the C-terminal helices, arranged in a zipper-like fashion, are crucial for substrate transport [11,12,13]. TmrAB, in the form of TmrA-TmrB heterodimer, mediates the update of Hoechst 33342 oriented in vesicles by hydrolyzing ATP. In this process, the canonical glutamate 523 site of TmrA is crucial for its conformational change from a ATP/ATP-bound to ADP/ATP state [14]. Additionally, by performing sequence alignment and biochemical assays, it has been demonstrated that the disruption of the GRD (a highly conserved region in intracellular loop 1) motif in TmrA results in different functional consequences than that in TmrB, suggesting that TmrA and TmrB likely carry out distinct functions in the conformational cycle of TmrAB [14,15]. Therefore, it is of interest to investigate the functions of the individual protein TmrA, in which efficient purification of this membrane protein is needed, and a comprehensive characterization of the protein–lipid complex would facilitate the optimization of the protein purification procedure. In our study, we focused on three nonionic detergents, DDM, Triton X-100 and OG, which are more likely to prevent protein denaturation and improve protein solubility. These detergents are mild nonionic detergents and help maintain membrane protein conformations. In previous studies, with respect to ABC transporters, purification workflow has been optimized, and DDM was the detergent of choice [11,16,17,18,19,20,21]. For example, in the study of BmrA purified from *B. subtilis*, a number of nonionic detergents and various concentrations of DDM were investigated for the optimal purification of BmrA, and BmrA was purified in a functional form in DDM [9].

Analytical ultracentrifugation (AUC) is a classic technique that characterizes the biophysical properties of biomacromolecules by recording their sedimentation behavior in solution. By measuring the sedimentation of membrane proteins in centrifugal fields, the molecular weight, purity, state of aggregation, and interactions of the assayed biological molecules can be deduced. Utilizing the UV/visible, interference or fluorescence detectors, AUC can be performed in two approaches: sedimentation velocity (SV) or sedimentation equilibrium (SE). With technical advances, AUC has been extensively applied to analyses of membrane proteins [7,22,23,24,25,26,27,28]. Using AUC, we discovered that TmrAB exists as a heterodimer of TmrA and TmrB in the solution containing 0.08% DDM, with a molar ratio of DDM/TmrAB equal to 116:1. This discovery demonstrates that AUC is capable of analyzing the molecular weight of membrane proteins [27]. In the current study, we aimed to optimize the purification procedure for TmrA, which paves the way for subsequent functional analyses and structural studies. TmrA is used as a model to demonstrate how the type and concentration of detergents impact the purification of membrane protein. Guided by AUC and electron microscopy (EM), we also proposed a cost-effective strategy to purify membrane proteins with less detergent followed by detergent supplementation after SEC chromatography. As a proof of principle, the protein aggregation state of TmrA acquired by this strategy is comparable to that acquired by the conventional method.

## 2. Materials and Methods

### 2.1. Expression and Purification of TmrA

#### 2.1.1. Expression of TmrA in *E. coli*

The cDNA sequence of TmrA with a His-tag at the C-terminus was cloned into the pET15D vector and transformed into *E. coli* BL21 (DE3) cells. Cells were cultured overnight at 37 °C and 220 rpm in 100 mL LB medium with 100 µg/mL ampicillin. An amount of 10 mL of the culture was transferred to 1 L of LB medium (1:100 dilution) and incubated in a shaker at 220 rpm and 37 °C to an OD_600_ of 1.0~1.2, which usually takes about 3–4 h. We added 0.5 mM IPTG to induce protein expression, and cells were incubated overnight at 20 °C and 220 rpm.

#### 2.1.2. Purification of TmrA in DDM

Cells were harvested by centrifugation at 4000 rpm (~4542× *g*) for 10 min at 20 °C. The cell pellet was refrigerated for immediate purification or stored at −80 °C. The cell pellet was resuspended in lysis buffer containing 25 mM Tris, pH 8.0, and 150 mM NaCl. After sonication on ice, cell lysates were centrifuged at 14,000 rpm (~27,000× *g*) for 10 min. Then, the suspension was centrifuged at 41,000 rpm (~194603× *g*) and 4 °C for 1 h to isolate the cell membrane. The resulting cell membrane was then resuspended in the lysis buffer supplemented with 1% (*w*/*v*) DDM (Anatrace, Maumee, OH, USA) and subsequently incubated for 2 h at 4 °C. After an additional incubation at 65 °C for 30 min, the sample was centrifuged at 41,000 rpm for 30 min.

The supernatant was incubated with Ni-NTA resin (QIAGEN, Hilden, Germany) at 4 °C for 30 min, and was washed 3 times with washing buffer containing 20 mM imidazole, 25 mM Tris, pH 8.0, 150 mM NaCl, and 0.02% DDM (2 CMC, the critical micelle concentration of DDM is 0.01%). TmrA was eluted with elution buffer containing 250 mM imidazole, 25 mM Tris, pH 8.0, 150 mM NaCl, and 0.02% DDM. SDS-PAGE was performed to validate the fractions containing the purified protein, and the sample was then subjected to size exclusion chromatography (SEC) with a Superdex 200 increase 10/300 GL (Cytia, Marlborough, MA, USA) for further purification. Superdex 200 Increase column was pre-washed and equilibrated with 24 mL buffer containing 25 mM Tris, pH 8.0, 150 mM NaCl, and 0.02% DDM. The protein sample was centrifuged for 5 min at 12,000 rpm, and then fractionated by the column with a flow rate of 0.5 mL/min. Peak fractions were collected, and SDS-PAGE was conducted for verification [28].

#### 2.1.3. Detergent Exchange

In order to evaluate the aggregation state of TmrA in different detergents, DDM was replaced with Triton X-100 (Amersco, Radnor, PA, USA) or OG (Anatrace, Maumee, OH, USA). During membrane extraction, 1% (*v*/*v*) Triton X-100 or 10% (*w*/*v*) OG was used in the lysis buffer. After incubation with Ni-NTA resin, TmrA was washed and eluted with buffer containing 0.03% Triton X-100 (2 CMC, the critical micelle concentration of Triton X-100 is 0.015%) or 1% OG (2 CMC, the critical micelle concentration of OG is 0.5%). Following Ni-NTA, SEC was conducted as described in Section 2.1.2, but DDM in all buffers was replaced with 0.03% Triton X-100.

### 2.2. Analytical Ultracentrifugation

Sedimentation velocity was performed with an Optima AUC (Beckman Coulter, Brea, CA, USA) equipped with an 8-cell An-50 Ti rotor at 20 °C. Analysis buffer (25 mM Tris, pH 8.0, and 150 mM NaCl) was used as the reference solution, and TmrA in different detergents was analyzed at the speed of 45,000 rpm (~163,296× *g*). Concentration profiles were recorded using UV absorption (280 nm) and interference scanning optics, and analyzed using SEDFIT (available at http://sedfitsedphat.nibib.nih.gov/software, accessed on 1 September 2021) and GUSSI (available at http://biophysics.swmed.edu/MBR/software.html, accessed on 1 September 2021). We used a continuous *c*(s) distribution model combined with prior knowledge to determine different species by diffusion coefficients. 

### 2.3. Negative Staining Electron Microscope

The carbon film coated with 300 mesh copper EM grids was subjected to glow discharger for 30 s. Then, 4 µL TmrA protein samples (0.01–0.02 mg/mL) were mounted onto the grid and incubated for 1 min at room temperature, and the excess solution was removed by filter paper. Samples were immediately stained with 3% uranyl acetate (UA) solution on the surface of the EM grid for 10 s. UA staining was applied 3 times with 2 min incubations, and samples were dried at room temperature and stored in an EM grid box.

A negative staining electron microscope study was performed using a FEI Tecnai Spirit TEM D1319 (FEI, Hillsboro, OR, USA). Prior to imaging at 120 KeV, TEM was aligned properly, and the defocus was optimized for imaging [29].

## 3. Results

### 3.1. TmrA Possesses Different Aggregation Status in Distinct Detergents

**Figure 1:**
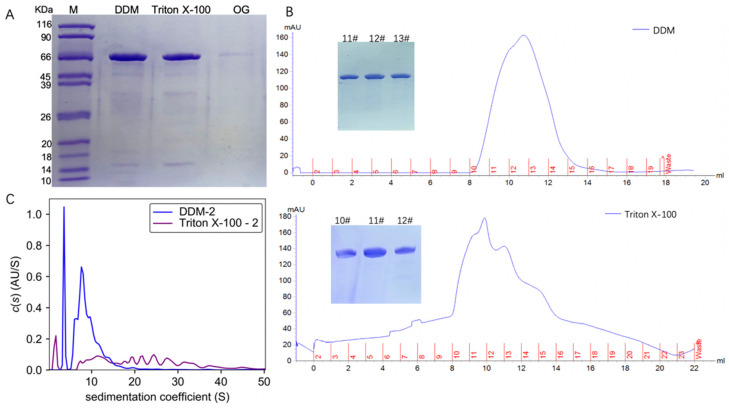
Characterization of TmrA after purification by different detergents. (**A**) SDS-PAGE analysis of the TmrA purification after Ni-NTA affinity chromatography. (**B**) Profiles of UV absorbance of size-exclusion chromatography and SDS-PAGE results (inlet) of the indicated fractions. (**C**) Overlay of *c*(s) distributions obtained for TmrA purified with 2 CMC DDM or Triton X-100 when analyzed by interference light detector of the AUC.

TmrA with a C-terminal His tag was expressed in *E. coli* BL21 (DE3) and purified by Ni-NTA affinity chromatography and size exclusion chromatography (SEC). TmrA bound to Ni-NTA was eluted with elution buffer containing 250 mM imidazole and 2 CMC detergents (DDM, Triton X-100, or OG). Following Ni-NTA chromatography, the eluates were analyzed by SDS-PAGE (Figure 1A). TmrA purified by 2 CMC DDM and Triton X-100 showed almost comparable yield and purity, whereas 2 CMC OG yielded much lower protein abundance. Despite extended elution, the yield of protein purified with 2 CMC OG was still insufficient for subsequent analyses.

The TmrA samples in DDM and Triton X-100 were subjected to SEC, respectively (Figure 1B), and the peak fractions were collected and analyzed by SDS-PAGE. According to the SDS-PAGE and UV absorbance, similar abundance and purity of TmrA were observed in 2 CMC DDM and Triton X-100. Following SEC, peak fractions from each sample were pooled and analyzed by the interference light detector of the AUC (Figure 1C). It is of note that Triton X-100 can absorb UV light, which interferes with the ultraviolet readings by SEC and AUC. Compared with SEC, AUC is capable of assessing more comprehensive physicochemical properties of the protein samples. By examining the sedimentation coefficient, it is apparent that 2 CMC Triton X-100 leads to a higher level of heterogeneity than DDM, with *s* spreading between ~7 and 40 S. In 2 CMC DDM, however, only two major peaks were observed, and the *s* is between ~5 and 20 S, indicating better homogeneity.

### 3.2. High Concentration of DDM Improves the Homogeneity of TmrA

With 2 CMC DDM, the UV absorbance displayed a wide distribution between 8 and 13 mL, indicating a high degree of aggregation (Figure 1B). To investigate the impact of detergent concentrations on the homogeneity and aggregation state of TmrA, we also performed purification with 6 CMC and 10 CMC DDM.

**Figure 2:**
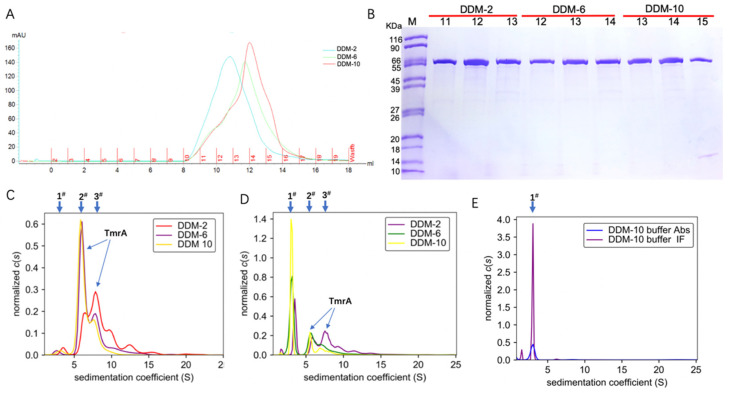
Characterization of TmrA behavior purified with different concentrations of DDM using SEC and SV-AUC. (**A**) Overlaid profiles of UV absorbance of TmrA samples in 2 CMC DDM (blue), 6 CMC DDM (green), and 10CMC DDM (red) by SEC. (**B**) SDS-PAGE analysis of fractions collected after SEC, DDM concentrations, and the fraction numbers are indicated above the respective lanes. (**C**) Overlay of *c*(s) distributions obtained for TmrA purified with DDM of 2 CMC, 6 CMC, and 10 CMC when analyzed by the ultraviolet/visible detector of the AUC. (**D**) *c*(s) distributions of TmrA purified with DDM of 2 CMC, 6 CMC, and 10 CMC when analyzed by the interference light detector; (**E**) *c*(s) distributions of 10 CMC DDM buffer when analyzed by the ultraviolet/visible and interference light detectors.

With an increasing concentration of DDM in the eluent, the TmrA eluates showed narrower peaks and higher elution volumes of ~10.8 mL, 11.8 mL, and 12.5 mL at 280 nm (Figure 2A). The increasing elution volumes suggest a reduction in protein aggregation, and the narrower absorbance peaks are indicative of improved homogeneity. Therefore, more homogeneous and well-behaved TmrA was acquired with a high concentration of DDM, and it yielded similar purity compared to lower concentrations, as indicated by the SDS-PAGE result (Figure 2B).

To substantiate our findings, we adjusted the concentration of protein eluates from SEC to 0.6 mg/mL and analyzed them by the ultraviolet/visible light and interference light detectors of AUC (Figure 2C,D). In addition, 10 CMC DDM buffer without protein was used as the blank control (Figure 2E). Based on the data acquired by the ultraviolet/visible light detector (Figure 2C), the TmrA in 2 CMC DDM showed a first peak (1^#^) of *s_20,w_* = 3.584 S, a major peak (3^#^) of *s_20,w_* = 8.052 S, and a minor peak (2^#^) of *s_20,w_* = 6.432 S, together with a number of smaller peaks of higher sw values representing protein aggregation. In contrast, the TmrA in 6 CMC DDM showed a first peak (1^#^) of *s_20,w_* = 2.747 S, a major peak (2^#^) of *s_20,w_* = 6.199 S, and a minor peak (3^#^) of *s_20,w_* = 8.849 S; the TmrA in 10 CMC DDM showed the first (1^#^), major (2^#^), and minor (3^#^) peaks of *s_20,w_* = 3.504 S, *s_20,w_* = 6.000 S, and *s_20,w_* = 7.899 S, respectively. The first peak (1^#^) is a result of the micelles of DDM (Figure 2C), as the 10 CMC DDM buffer displays a similar peak (1^#^) of *s_20,w_* = 2.968 S (Figure 2E). Similar results were obtained from the interference light detector. According to the in-depth analysis by AUC, the second peak (2^#^) represents TmrA monomer (labeled in Figure 2C,D), and the detailed analyses of the second peak (2^#^) are shown in Table 1. These results suggest that DDM impacts the aggregation state of TmrA in a concentration-dependent manner, and TmrA is more homogeneous in 10 CMC DDM, in agreement with the SEC data.

### 3.3. Detergent Supplementation Is a Cost-Effective Strategy and Does Not Compromise Protein Homogeneity or Aggregation State

**Figure 3:**
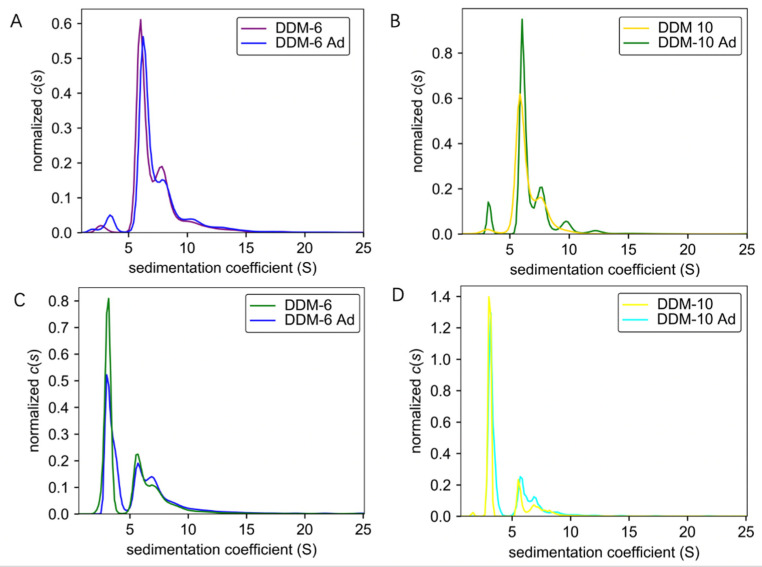
Comparisons of sedimentation coefficients of the TmrA samples purified with constant DDM concentration or DDM supplementation. *(***A**,**B**) Pairwise comparisons of *c*(s) distributions obtained for TmrA purified with 6 CMC DDM vs. 6 CMC DDM-Ad (**A**) and 10 CMC DDM vs. 10 CMC DDM-Ad (**B**) when analyzed by absorbance detector of the AUC. (**C**,**D**) Pairwise comparisons of *c*(s) distributions obtained for TmrA purified with 6 CMC DDM vs. 6 CMC DDM-Ad (**C**) and 10 CMC DDM vs. 10 CMC DDM-Ad (**D**) when analyzed by interference light detector of the AUC.

It is well established that detergents are crucial for the purification of membrane proteins. However, the cost of detergents has been a daunting consideration. The successful purification of TmrA requires a high concentration of DDM, which prompted us to pursue a more cost-effective strategy for protein purification. To further optimize the purification procedure, we investigated whether supplementing the protein samples in a low concentration of DDM with DDM after SEC, which would significantly reduce the use of detergent in early steps, would achieve a comparable protein homogeneity and aggregation level. Protein samples purified in 6 CMC DDM and 10 CMC DDM were obtained by either using the corresponding concentrations of DDM throughout the purification process or adding 100 CMC DDM to samples purified by 2 CMC DDM to achieve a final concentration of 6 or 10 CMC. All the protein samples were adjusted to 0.6 mg/mL and subjected to AUC. By combining analyses by ultraviolet/visible (Figure 3A,B) and interference (Figure 3C,D) light detectors, the sedimentation coefficients of samples purified by constant concentrations of DDM (DDM-6 and DDM-10) and those of DDM supplementation (DDM-6 Ad and DDM-10 Ad) largely overlap. These results suggest that the addition of detergent after SEC does not compromise the homogeneity or aggregation status.

### 3.4. Negative Stain Electron Microscope Defines Protein Aggregation States of TmrA Purified by the Optimized Workflow

**Figure 4:**
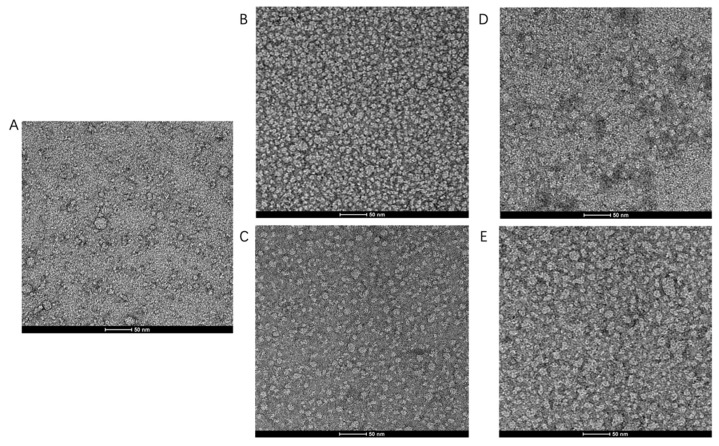
Raw images of TmrA samples by negative stain EM. Raw negative stain EM images of TmrA purified with 2 CMC DDM (**A**), 6 CMC DDM (**B**), 10 CMC DDM (**C**), 6 CMC DDM-Ad (**D**), and 10 CMC DDM-Ad (**E**).

In order to substantiate our findings by AUC, images of negative stain electron microscopy (EM) were acquired to assess the monodispersity and oligomeric state of TmrA (Figure 4). Heterogeneous and aggregated TmrA particles were observed in 2 CMC DDM (Figure 4A), consistent with our findings by SEC and AUC (Figure 2 and Table 1). The samples purified in 6 CMC and 10 CMC DDM displayed homogeneous states and dispersed evenly (Figure 4B,C). Next, we examined whether supplementing 2 CMC DDM with a high concentration of DDM could remedy the heterogeneity. Indeed, the addition of DDM could reduce the heterogeneity and protein aggregation (Figure 4D–E), which resembles the samples purified in a constant concentration of DDM for both 6 CMC and 10 CMC DDM (Figure 4B,C). These images agree with our findings resulting from SEC and AUC, suggesting the effectiveness of this detergent supplementation strategy.

## 4. Discussion

In this study, we purified the membrane protein TmrA from *E. coli* using Ni-NTA and SEC, and we analyzed its aggregation status using AUC and EM. The results suggest that the choice of detergent and the concentration of detergent dictate the behavior of TmrA. Additionally, we verified a cost-effective method to purify TmrA, which may facilitate membrane protein purification.

A prominent barrier to studying membrane proteins is the solubilization and purification from their native conditions. The application of diverse detergents, such as ionic detergents, bile acid salts, nonionic detergents and zwitterionic detergents, has tremendously accelerated membrane protein purification. Nonionic detergents are generally mild and nondenaturing, without affecting the native conformations of membrane proteins [30,31]. Due to their amphiphilic nature, these mild detergents can improve the solubility of membrane proteins. In our study, the three detergents tested (DDM, Triton X-100, and OG) all belong to mild nonionic detergents. *N*-octyl-β-d-glucopyranoside (OG) has a short chain (C_7_), which may lead to protein denaturation and deposition on Ni-NTA resin. This may explain the lower yield of TmrA by Ni-NTA affinity chromatography. Triton X-100, which has an intermediate C_9_ chain, can effectively extract TmrA from its native biolayers. However, the non-negligible ultraviolet absorbance of Triton X-100 interferes with the analyses by SEC and AUC. N-dodecyl-β-d-maltopyranoside (DDM), with a longer chain (C_11_), has seen increasing implementation in membrane protein solubilization. DDM can efficiently purify TmrA as well as Triton X-100, without interfering with UV absorbance; thus, DDM is preferred for TmrA purification. It is also noteworthy that the concentration of detergent can influence protein purification, particularly the aggregation state of the target protein [9]. In the current study, a high concentration of DDM led to less aggregation and higher homogeneity of TmrA.

Analytical ultracentrifugation is a powerful approach to assessing membrane protein behavior in a quantitative manner. As SV-AUC can characterize protein aggregation states, it may provide insights into the appropriate concentration of detergent in membrane protein purification. Specifically, by combining absorbance and interference detections, different states of membrane proteins in solution can be distinguished by their sedimentation coefficients in sedimentation velocity [32]. The *c*(s) curves reflect the compositions of the assayed biomolecules in solution. Although SE-AUC is particularly instrumental in determining molecular weights of membrane proteins dispersed in detergent micelle solution [9,33], its application requires preliminary experiments on protein samples performed at different concentrations and a series of centrifugation speeds, which may take days to accomplish. Therefore, we only performed SV-AUC analyses in this study in the interest of time.

Altogether, our results suggest that DDM is preferred for purifying TmrA from the bacterial membrane, and the aggregation state of TmrA is linked to the concentration of DDM. Additionally, the sample purified in 10 CMC DDM is more homogeneous than that in 6 CMC or 2 CMC DDM. Moreover, the addition of DDM after SEC improves the homogeneity of the samples without compromising the protein aggregation state. It is noteworthy that the experimental procedure and buffers we used are in line with previous investigations [8,14,17,20,21], so extensive protein aggregation or misfolding were not noticeable in our experiments. Even though the DDM supplementation results in *c*(s) distributions overlapping with a high concentration of DDM, it still remains to be experimentally determined whether TmrA possesses similar activity.

Here, we propose a cost-effective procedure to purify membrane protein TmrA based on the meticulous examination of protein behavior in various conditions, which highlights the contribution of AUC to characterizing protein behavior during sample preparation. In addition, our study provides insights into how to optimize protein purification by employing AUC. Although our study is limited to TmrA, given the similarity in sequence and structure in the ABC transporter family found in prokaryotes and eukaryotes, we anticipate that this study will be instrumental for the purification of ABC transporters and will be of interest for a broader community that investigates membrane proteins. Given its capability to characterize protein–lipid complexes, we envision extensive implementation of AUC in assisting structural biology, biomedical, and biophysical studies.

## Figures and Tables

**Table 1:** Parameters regarding TmrA aggregation state derived from SV-AUC analyses.

Peak Data	Units	DDM-2	DDM-6	DDM-10
MW ^1^	KDa	64.6	64.6	64.6
Mp ^2^	KDa	52.3	66.6	64.2
f/f_0_		1.26	1.37	1.335
delta D ^3^		157 ± 7	135 ± 6	117 ± 5
		**Abs/IF ^4^**		
*s* *_20,w_*	S	6.4/6.1	6.2/5.9	6.0/5.7
proportion of total	%	22.7/18.4	61.3/26.8	56.7/10.8
Stokes radius	nm	3.39/4.41	3.50/5.21	3.48/4.75
a/b (oblate)		1.47/5.03	2.12/8.00	2.23/6.72
a/b (prolate)		1.45/4.69	2.07/7.23	2.18/6.15

^1^ Theoretical molecular weight calculated from protein sequence. ^2^ Molecular weight derived from AUC data analyzed by Sedfit and GUSSI software. ^3^ Detergent/protein molar ratio. ^4^ Absorbance/Interference of peak intensity.
